# Genetics, Pharmacokinetics, and Neurobiology of Adolescent Alcohol Use

**Published:** 2004

**Keywords:** adolescent, AOD (alcohol and other drug) use initiation, alcohol abuse, alcohol dependence, AOD sensitivity, use initiation, risk factors, protective factors, genetic risk and protective factors, heredity factors, genetics and heredity, environmental factors, heredity vs. environmental factors, human study, twin study, animal study, animal model, puberty, biological development, psychological development, cognitive development, endocrine system, hormones, stress, ethanol metabolism, alcohol metabolism, pharmacokinetics

## Abstract

Complex behaviors such as the initiation and use of alcohol result from an intricate interplay between genes and environment. Genes shape physiological and behavioral responses to alcohol that can influence the likelihood that a young person will begin using alcohol and that he or she will progress to problem drinking. Youthful alcohol use also can have an impact on unfolding developmental patterns, and for some, early use becomes the entry point for pathways that lead to problems with alcohol. This article first describes research on genes that may be involved in the development of alcohol problems and how genetic factors may contribute to adolescent alcohol use. It then examines how the changes that occur during adolescent development—in alcohol metabolism, in the brain, and in the endocrine and stress response systems—may affect how a young person experiences alcohol and the likelihood that he or she will develop alcohol use problems.

## Overview

Many studies have focused on how physiological and neurobiological responses to alcohol— such as sensitivity to alcohol, change in its rewarding effects, craving, tolerance and withdrawal—factor into the development of alcohol use and alcohol use disorders. The majority of studies to identify genes and neurobiological mechanisms that may contribute to alcohol use and alcohol use disorders in humans have been done with adults. Recent data suggest, however, that the highest prevalence of alcohol dependence in the general population occurs in people ages 18–24, and it is not yet clear to what extent genes involved in the onset of alcohol problems in adults play a similar role in youth. A central goal of research is to understand the genetic and environmental factors, and the interplay between them, that contribute to the development of alcohol abuse and dependence in adolescents.

Human genetic research related to alcohol use has involved studies with twins or with families that have a high prevalence of alcohol-dependent individuals. This work has identified regions of chromosomes that are associated with an altered risk of developing alcohol dependence, and in some cases, individual genes or candidate genes. Analysis of the role these genes and gene regions play in alcohol use is difficult for a number of reasons. As with other complex genetic diseases, multiple genetic factors may contribute to the risk of developing alcohol dependence, but no one factor is associated with a large percent of risk. Different risk factors may be active in different individuals. In the case of alcohol use, studies of both adults and adolescents suggest that the relative contributions of genes and environment change at different stages of problematic drinking; for example, genes have a strong influence over the development of problem use, whereas environment seems to play a greater role in the initiation of alcohol use.

Research using animal models also added to our understanding of the genes that may contribute to alcohol abuse and/or dependence. Animal studies have helped to identify genes involved in the effects of alcohol as well as those involved in the pathways affecting sensitivity to acute alcohol exposure, reward, craving, and withdrawal. Many of the genes that have emerged from this research have roles in other behaviors as well. For example, serotonin has been implicated in alcohol consumption in conjunction with its role in anxiety, which, in some individuals, is particularly manifest during adolescence. Further studies will assess the relative roles of various signaling pathways and their component genes in the development of alcohol-related behavior, and determine which are most influential in adolescents.

Superimposed on genetically shaped aspects of physiology are developmental changes that can measurably affect both the response to alcohol and the chances that someone will drink heavily. Some research, for example, suggests modest changes during adolescence in the pharmacokinetics of alcohol—how it is absorbed, distributed, and eliminated. Gender differences in these processes emerge during puberty and affect blood alcohol levels after drinking.

Animal studies suggest that sensitivity to alcohol is different among adolescents than it is in adults. For example, adolescent rats are less sensitive to the unpleasant effects of intoxication, such as sedation, loss of coordination, and hangover effects, and they consume higher levels of alcohol than do older animals. Possible underlying reasons for the lack of sensitivity include the developmental immaturity of neurotransmitter receptor systems.

Dramatic hormonal changes take place during puberty, affecting growth and sexual development, and the body’s stress response systems. These hormonal changes also may affect sensitivity to alcohol. For example, animal research suggests that hormones involved in the response to stress may interact with neurotransmitters in the brain that are associated with the sensation of reward to facilitate drinking.

Clearly, development adds a layer of complexity to understanding the reciprocal interactions between genes and environment, alcohol metabolism, and the neurobiology of the response to alcohol.

## Genetics of Adolescent Alcohol Use and Alcohol Use Disorders

Accumulating research indicates that complex behaviors result from the interplay between genes and environment over developmental time. Alcohol use is a prime example of a complex behavior in which gene expression and environment/context reciprocally influence one another. For example, during adolescence, biological and physiological changes may promote risk-taking behavior, thereby influencing the way people decide to spend their time. The environments that a person selects may foster the use of alcohol, which in turn may result in acute physiological reactions that have the potential to trigger long-term biological changes. These changes then may affect the person’s more immediate behavior as well as move unfolding developmental pathways toward adverse outcomes, including psychopathology (e.g., anxiety disorders). In this way, youthful patterns of alcohol initiation and escalation of use can become entry points for pathways that ultimately lead to abuse and dependence.

However, not all young people experience the same outcome(s) from what may appear to be similar patterns of adolescent alcohol use. Studying such complexity is difficult, and adding to the difficulty of understanding these complex pathways at the biological level is the recognition that, unlike other drugs of abuse, alcohol does not appear to act through a specific receptor. Instead, alcohol modulates the function of multiple neurotransmitter systems and voltage-gated ion channels.

At the macro level, pathways that have been associated with the development of alcohol problems in general, and alcohol dependence more specifically, manifest themselves in the form of multiple behavioral and physiological characteristics, including disinhibition/impulsivity, anxiety, variations in the intensity of response to alcohol, and several independent psychiatric disorders and alcohol-metabolizing patterns. These characteristics can influence the development of alcohol dependence through alterations in pathways that determine the rewarding effects of alcohol, tolerance to some of its intoxicating effects, pathologic effects on the brain, and the development of withdrawal symptoms (Koob and Le Moal 1997). Genetically conferred characteristics contribute to the degree and/or rate of development of these changes (Crabbe 2001).

Attempts have been made to categorize people based on the degree and rate that they develop dependence and to differentiate between the genetic contributions to various categories of alcohol dependence (Cloninger et al. 1981; Babor et al. 1992). Because the development of alcohol use, abuse, and alcoholism occurs on a continuum, and because recent data show that the highest prevalence of alcohol dependence in the population occurs between the ages of 18 and 25, this review focuses on efforts to identify genes contributing to those pathways that appear in childhood and adolescence (Grant et al. 2004). It must be emphasized, however, that many of the studies identifying heritable components which may contribute to alcohol abuse or dependence have been carried out in adults. The extent of their role, if any, in the development of alcohol problems in youth, therefore, remains to be determined. Additional genetic contributors to alcohol problems may be identified by future investigations, and some of these may be more specific to risk in children and adolescents.

Finding genes that contribute to the development of alcohol abuse and dependence in humans may be simplified by focusing on endophenotypes (i.e., intermediary connections between the manifestation of dependence and its biological underpinnings). To this end, human genetic studies have focused on families that have a high prevalence of members with alcohol dependence, on twin studies, and on studies assessing potential markers and/or contributors to risk. By performing genetic analyses on such populations, researchers have identified specific regions on individual chromosomes that correlate with risk for alcohol dependence (e.g., Reich et al. 1998; Long et al. 1998; Foroud et al. 2000). In some cases, individual candidate genes have been associated with these regions.

The goal now is to further refine those regions for which a specific gene has not yet been identified as responsible for the observed phenotype and to determine respective contributions of candidate genes to alcohol dependence. Research on how the identified genes interact with other genes and gene products and with the environment to result in alcohol dependence also is important. To date, functional polymorphisms in the alcohol-metabolizing enzymes alcohol dehydrogenase and mitochondrial aldehyde dehydrogenase have been the most thoroughly documented for providing protective effects in specific populations (reviewed in Oroszi and Goldman 2004). In addition, the Collaborative Study on the Genetics of Alcoholism (COGA) has identified specific genes within identified regions that affect risk for alcoholism (reviewed in Edenberg and Kranzler 2005). These include gamma-aminobutyric acid (GABA) receptor subunits GABRA2 and GABRG3 and the muscarinic cholinergic receptor. GABRA2 has been independently confirmed (reviewed in Edenberg and Kranzler 2005).

Other promising candidates that have been implicated and are under investigation include the serotonin transporter 5-HTT (reviewed in Oroszi and Goldman 2004); specific alleles of the neurotransmitter dopamine (reviewed in Bowirrat and Oscar-Berman 2005); catechol*-O-*methyltransferase (COMT) (reviewed in Oroszi and Goldman 2004); neuropeptide Y (NPY) (reviewed in Oroszi and Goldman 2004; Mottagui-Tabar et al. 2005); and the μ opioid receptor (OPRM) (reviewed in Oroszi and Goldman 2004; Edenberg and Kranzler 2005; Bart et al. 2005).

### Animal Studies

In addition to studies with humans, studies using animal models, such as worms, flies, and rodents, permit researchers to model, in less complex systems, individual biological and behavioral components that may factor into alcohol problems, including dependence. The goal of this work is the convergence of findings from human and animal model studies to facilitate the design of pharmacological agents that can reduce, prevent, or ameliorate alcohol problems, including dependence and associated consequences.

In model organisms, several basic approaches have been used to uncover genetic influences. One approach is to generate inbred strains of animals (usually rodents) that voluntarily consume large amounts of alcohol and those which do not, and then perform genetic analyses to determine the gene(s) underlying this behavior (reviewed in Crabbe et al. 1994).

For example, a well-studied rodent model of alcoholism uses rats selectively bred for increased alcohol consumption (i.e., alcohol preferring [P] rats and nonpreferring [NP] rats). P rats, in addition to voluntarily consuming approximately 10 times more alcohol than NP rats, also self-administer alcohol and are willing to work for alcohol (reviewed in Crabbe et al. 1994). Because these characteristics are not typical of most rats, they indicate that, compared with NP rats, P rats possess a genetically determined difference in the neural substrate for determining the rewarding value of alcohol. These and other high-drinking strains of rodents are being used to identify chromosomal locations that correlate with the observed drinking behaviors and then to further refine this correlation to a single gene (e.g., for review in rats, see Bice et al. 1998; Carr et al. 2003; in mice, see Belknap and Atkins 2001). Although perhaps less complex than human genetic mapping, such research strategies remain complicated. And such searches are made even more difficult by the pleiotropic nature of genes (i.e., genes have multiple influences and sites of action) and their complex interactions.

Transgenic mice (i.e., mice that lack a specific gene [knockout mice] or overexpress a gene product in a specific cell, tissue, or region) also have been used to assess the role of individual genes. Examples of candidate genes that have been identified or confirmed using transgenic mice include neuropeptide Y (reviewed in Thiele et al. 2003), protein kinase A (Thiele et al. 1998), and the cannabinoid receptor1 (CB1; Wang et al. 2003). More recently, gene mapping has been combined with gene profiling (i.e., analysis and comparison of gene expression patterns to identify candidate genes within chromosomal regions). Recently, studies combining these lines of investigation have identified α-synuclein as a potential contributor to alcohol preference in inbred preferring (or iP) rats (Liang et al. 2003).

Another major approach using model organisms exploits the ability to generate mutations randomly across the entire genome of an organism (e.g., in worms or fruit flies) and then to screen these mutations for specific phenotypes or behaviors following alcohol administration. Because the basic behavioral responses to acute alcohol exposure are similar among humans, rodents, and flies, identification of mutant animals that are either more or less sensitive to alcohol exposure has been an area of active research. Studies in animals and humans suggest that reduced sensitivity to alcohol in an individual predicts development of alcoholism (Crabbe et al. 1994; Schuckit 1999).

Mutated flies that show an increased sensitivity to the acute effects of ethanol have been generated. Analyses of the gene mutations present in these flies have implicated the cyclic AMP (cAMP) signal transduction pathway in the regulation of acute ethanol sensitivity (Moore et al. 1998). The requirement for proper cAMP signaling then was mapped to a small group of neurosecretory cells in the fly brain, which led to the identification of a cluster of cells in this location that produces insulinlike peptides (DILPs) (Brogiolo et al. 2001; Rulifson et al. 2002). Within these specific cells, inhibition of protein kinase A (PKA), a downstream component of the cAMP signal transduction pathway, increased ethanol sensitivity. In addition, flies that have a mutation which causes a reduction in insulin receptor activity show increased ethanol sensitivity (Corl et al. 2005). These results suggest a role for the insulin-signaling pathway in regulating behavioral responses to alcohol. In apparent contradiction, flies with a mutation in pka-RII, one of the PKA regulatory subunits, showed reduced sensitivity to ethanol (Park et al. 2000). Similar to the pka-RII-deficient flies, mice lacking the regulatory subunit of PKA showed increased voluntary alcohol consumption and were less sensitive to alcohol’s sedative effects (Thiele et al. 2000). However, mice with reduced levels of Gα’s, the adenylyl cyclase–stimulating G-protein, show increased ethanol sensitivity and reduced voluntary consumption (Wand et al. 2001). As a group, these results illustrate the complex nature of the regulation of ethanol sensitivity by the cAMP signal transduction pathway in a variety of cell types.

Similar lines of investigation in flies and mice also have implicated neuropeptide Y (NPY) in mediating sensitivity to ethanol sedation and in modulating alcohol consumption (reviewed in Thiele et al. 2003; Wen et al. 2005). As mentioned previously, NPY also has been identified in human studies. In addition to sensitivity to acute alcohol administration, other factors such as reward, craving, and withdrawal also contribute to alcohol dependence. Both corticotropin-releasing factor (CRF) (reviewed in Valdez and Koob 2004) and NPY may play a role in maintenance of heavy drinking despite serious negative consequences. In addition, the dopamine pathway has been intensively studied to determine its role in the reward pathway (reviewed in Koob and Le Moal 1997). More recent studies also suggest a role for the endocannabinoids in the rewarding effect of ethanol (Wang et al. 2003).

Studies analyzing mutagenized worms have uncovered a role for the calcium-activated large conductance BK potassium channel in ethanol sensitivity (Davies et al. 2003). A primary function of this channel is to repolarize active neurons, so activation in the presence of ethanol would inhibit neuronal activity.

### Adolescence

The recognition that the prevalence of alcohol dependence is highest in those ages 18 to 24 focuses attention on identifying the genetic contribution to alcohol initiation in childhood and adolescence; adolescent developmental pathways to alcohol dependence; and adolescent vulnerabilities to the consequences of alcohol abuse.

Very early starters (those who initiate alcohol use before age 12) often have several co-occurring problems that may include various externalizing and internalizing behaviors. Ongoing research is attempting to determine the order of causation and the potential underlying mechanisms that may be responsible for multiple problems as well as to identify early markers that might indicate some of those in need of early intervention. For example, what Cloninger (1987) classified as Type II alcoholism (characterized as male-limited, early onset, and occurring in sensation-seeking people and people with impaired impulse control) historically has been attributed to serotonergic dysfunction (Linnoila et al. 1994). Variation in the serotonin transporter gene promoter (5-HTTLPR) in humans has been associated with Type II alcoholism as well as with other neuropsychiatric diseases (Hallikainen et al. 1999; Saunder et al. 1998). Work in nonhuman primates has confirmed this relationship between excessive alcohol consumption and serotonin system genes in macaques; and, in addition, it has integrated research on the effects of early environmental stressors. In particular, this work has shown how environmental factors can influence gene expression to produce different patterns of behavior (see also Barr et al. 2004 for review).

**Figure:**
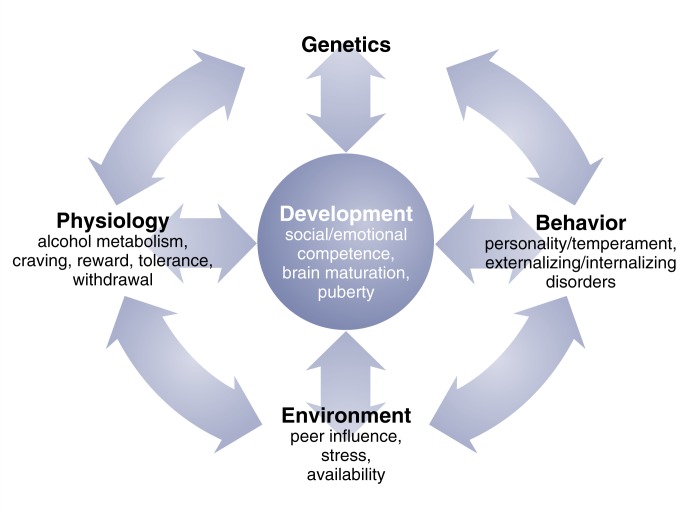
Alcohol use is a complex behavior that emerges from the interplay of genes and environment in the context of development.

The gene that encodes the enzyme monoamine oxidase A (MAOA) also influences synaptic concentrations of serotonin. Among adolescent and young adult male rhesus macaques, studies suggest that MAOA gene promoter variation may confer risk for alcohol dependence. Given that this gene resides on the X chromosome (males only have a single copy and females have two), males may be particularly susceptible to alterations in its expression. In humans, a polymorphism in the transcriptional control region of this gene has been associated with antisocial behavior in alcohol-dependent males (Samochowiec et al. 1999) and with impulsivity, hostility, and a lifetime history of aggression in a community sample of men (Manuck et al. 2000).

Environmental factors and genetic variations may result in similar phenotypes by affecting levels of specific gene products. For example, nonhuman primates that are removed from their parents at birth and reared with age-matched peers exhibit higher levels of anxiety and deficits in impulse control and are prone to violently aggressive behaviors. These animals also consume significantly higher volumes of alcohol and are more likely to drink to intoxication than are those reared with their parents under baseline nonstressful conditions (Higley et al. 1991). When stress is induced in mother-reared monkeys, however, they increase their alcohol consumption to that of their peer-reared counterparts. If peer-reared monkeys are given the serotonin reuptake inhibitor sertraline, alcohol consumption as well as anxiety and aggression are decreased (Higley and Linnoila 1997a). The level of the serotonin metabolite 5-hydroxyindoleacetic acid (5-HIAA) concentrations in the cerebrospinal fluid predicts subjects’ response to sertaline, suggesting that a deficit in serotonin underlies these behaviors (see also Barr et al. 2004 for review).

Consistent with the findings from adult twin studies, problematic use of alcohol in adolescence has been found to be more heritable than are initiation and more limited use. In a study specifically addressing adolescents, Rhee and colleagues (2003) applied a logistic regression model to data collected from sibling/twin/adopted adolescents ages 12–19. Their analysis resulted in the following conclusions:

- Alcohol initiation arises from genetic and from shared (common, family) and nonshared (uniquely experienced) environmental contributions.
- Alcohol use is minimally influenced by genetics but rather arises largely from environmental influences (shared, nonshared, and twin-shared experiences).
- Problem use of alcohol has a high heritability component and is significantly influenced by nonshared environmental influences (peer experiences, accessibility), but the contribution of shared environment (family) is small (Rhee et al. 2003).

Whether the specific genes underlying the stages in the progression from initiation to problem alcohol use are different, and whether the influence of specific genes and their expression vary with age and/or context remains to be determined.

These findings are consistent with those from other studies of adolescents. Rose and colleagues (2001) also reported that the strength of genetic influence on adolescent drinking behavior appears to increase from modest levels in midadolescence to moderate levels by late adolescence. And these types of findings are not unique to alcohol use; in late adolescence, genetic influences on problem drinking appear to overlap extensively with genetic influences on other indicators of disinhibited behavior (Krueger et al. 2002; Young et al. 2000). Although this latter research suggests that abusive drinking in late adolescence may be driven substantially by inherited differences in a general disposition to undercontrolled behavior, it does not rule out the influence of alcohol-specific genetic effects (i.e., genetic influences on alcohol sensitivity), or the impact of contextual factors. Indeed, mounting evidence exists that genetic influences on complex behavioral outcomes such as drinking behavior reflect a complex interplay between inherited and environmental factors (Rutter and Silberg 2002), the implications of which are only beginning to be explored for models of adolescent drinking (Rose et al. 2001).

## Adolescence and in Vivo Alcohol Pharmacokinetics

Adolescence is associated with profound physiological changes that almost certainly have an impact on in vivo alcohol pharmacokinetics. There is scant direct evidence in humans of any differences between the pharmacokinetics of alcohol in adolescents compared with adults, and for good reason—no one would expose young people to alcohol for research purposes. Although data relevant to this point in humans is lacking, the ontogeny (i.e., development over the life span of an individual) of ethanol metabolism has been examined in laboratory animals. (Note, however, that what may be called “adolescence” in animals may not precisely correspond to the same developmental period in humans, especially when differences in the timing of adolescence in human males and females are considered.) These studies suggest general ontogenetic increases in alcohol dehydrogenase activity (Raiha et al. 1967; Lad et al. 1984), ethanol elimination rates (see Kelly et al. 1987), and the rate of ethanol metabolism (Silveri and Spear 2000). An exception to this pattern sometimes has been seen during adolescence, with adolescent animals occasionally reported to exhibit slightly higher levels of ethanol metabolism than more mature animals (Hollstedt et al. 1977; Brasser and Spear 2002). Significant elevations in ethanol metabolism during adolescence are not always evident (e.g., Kelly et al. 1987; Silveri and Spear 2000), however, and are insufficient to account for the attenuated sensitivity to certain ethanol effects seen in adolescent animals relative to their more mature counterparts (see Little et al. 1996; Silveri and Spear 2000). What can be expected in human adolescents with respect to specific aspects of pharmacokinetics as they relate to alcohol effects is based on inference from animal studies and from research on adults, elements of which are described in the following sections.

### Distribution Volume

One feature of puberty is the appearance of gender differentiation in body fat. Girls have increased fat as a percentage of body weight (BWt). Because ethanol is soluble in water, the distribution volume for ethanol (V_D_, water space/BWt) is decreased in girls. Thus, girls experience higher blood alcohol concentrations (BACs) when they receive an ethanol dose that is proportionate to BWt (g ethanol/Kg BWt). In contrast, boys typically gain muscle mass and lose fat, increasing V_D_ and thus reducing the BAC reached after they receive an ethanol dose proportionate to body weight (NIAAA 1993).

### Elimination of Alcohol

Women reportedly metabolize alcohol faster than do men, a difference that probably becomes evident over the period of pubertal development. Similarly, the reported variation in alcohol elimination associated with the menstrual period among women presumably develops over the same age range. Whether the gender difference is an effect of changes in males or females (or both) should be determinable in animals (NIAAA 1993).

### Absorption Rate and Bioavailability

Both the rate of alcohol absorption and bioavailability are largely influenced by prandial state (the quantity—and perhaps quality—of food recently ingested). Gender differences in this interaction, particularly as they may affect first-pass metabolism (the metabolism of alcohol in the stomach and its first passage through the liver), have been suggested by some but not all studies in adults. Nothing is known about pre- vs. post-pubertal changes (NIAAA 1993).

## Neurobiological Mechanisms of Adolescent Alcohol Abuse and Dependence

Over the past 10 years, basic human and animal research has generated important new knowledge in the following areas: (1) identification of neurobiological and behavioral risk factors for alcohol abuse and dependence; (2) determination of the consequences of acute and chronic heavy drinking during adolescence on brain and behavioral maturation; (3) understanding of the neuropharmacological, neuroanatomical, hormonal, and behavioral mechanisms underlying the variable response to alcohol across developmental stages; and (4) assessment of the contribution of early alcohol exposure (during juvenile and adolescent periods) to excessive drinking and abnormal cognitive and social functioning in adulthood. Below is a summary of the current research findings on the neurobiological mechanisms involved in adolescent drinking.

### Predisposition to Alcoholism

Neurobehavioral research in human adolescents has largely been limited to studies of neural risk markers in children with a positive family history of alcoholism. These investigations suggest that there are subtle heritable neurocognitive and neurophysiological abnormalities in children of recovering alcoholics which could be early indicators of risk for alcoholism (see Tapert and Schweinsburg 2005 for a review). The most common finding is reduced P3 amplitude of the event-related potential in children with familial alcoholism (Begleiter et al. 1984; Hill and Steinhauer 1993). More recently, other neural risk factors that predate the onset of heavy drinking are being considered in at-risk youth, such as sleep electroencephalographic abnormalities (Dahl et al. 2003) and changes in brain structure (Hill et al. 2001) and function (Schweinsburg et al. 2004). For example, it was found that youths with dense family histories of alcoholism show reduced right amygdala volumes, which correlate with P3 amplitudes (Hill et al. 2001). More importantly, the neurophysiological and neuroanatomical abnormalities may be most pronounced during the prepubertal and adolescent years. This latter finding underscores the importance of considering developmental phases when attempting to identify early risk markers for alcoholism.

Taken together, these studies indicate that subtle neural abnormalities may underlie the heritable aspects of alcohol use disorders (AUDs) (Begleiter and Porjesz 1999; Pihl and Peterson 1996). However, some studies suggest that family history of AUDs primarily affects brain functioning in people who also show conduct disorder, antisocial personality disorder, sensation seeking, behavioral undercontrol, difficult temperament, or poor impulse control (Bauer and Hesselbrock 1999*a*,b; Schuckit 1998; Schuckit and Smith 1997; Tarter et al. 1985). Understanding these brain characteristics helps us to appreciate the brain abnormalities that may be produced by personal alcohol involvement as opposed to features that are attributable to predrinking risk factors.

As discussed in the genetics section, animal models have been used to study heritable factors that contribute to alcoholism. The selectively bred alcohol-preferring (P) and high alcohol drinking (HAD) lines of rats are particularly good models for studying the neural mechanisms of early onset drinking because they readily consume alcohol in the postnatal weaning stage and attain adult levels of intake by adolescence. Even as early as adolescence, innate differences are observed in the P and HAD lines in several neurobiological markers that have been associated with a genetic susceptibility to high alcohol drinking (McKinzie et al. 1998, 2002; Strother et al. 2003; see McBride et al. 2005 for review).

Further, nonhuman primates with low levels of the serotonin metabolite 5-HIAA have been used to model key aspects of adolescent behavior, such as impulsiveness and aggressiveness, tolerance to alcohol’s effects on initial exposure to alcohol, and the ability to drink excessive amounts of alcohol (Higley et al. 1996). Increased availability of serotonin transporters and low platelet monoamine oxidase activity also are thought to be traitlike markers in non-human primates associated with alcohol sensitivity and increased alcohol consumption (Heinz et al. 2003; Fahlke et al. 2002).

This pattern of behavioral and biochemical markers is similar to that predisposing to early onset alcoholism in humans and is influenced by genotype–environment interactions. For example, recent studies in rhesus monkeys found that serotonin transporter genotype influences cerebrospinal fluid 5-HIAA levels as well as alcohol sensitivity, preference, and consumption, but only in animals exposed to early life stress (Bennett et al. 2002; Barr et al. 2003). Thus, it is important to understand the relationships among environmental factors, genetic backgrounds, and neurobiological markers in predisposing an individual to alcoholism.

### Ontogeny of Initial Tolerance and Sensitivity to Alcohol

Adolescent rats consume higher absolute levels of alcohol than do older animals as a result of multiple factors. One is that adolescents are less sensitive than adult animals to the aversive effects of acute intoxication (e.g., sedation, ataxia, social impairment, and acute withdrawal/hangover effects) (Little et al. 1996; Silveri and Spear 1998; White et al. 2002; Doremus et al. 2003; Varlinskaya and Spear 2004; for review, see Spear 2000 and Spear and Varlinskaya 2005). Another is their greater sensitivity to alcohol-induced social facilitation and stimulation of alcohol intake by social experiences (Hunt et al. 2001; Varlinskaya et al. 2001; Varlinskaya and Spear 2002; for review, see Spear 2000 and Spear and Varlinskaya 2005). The neural basis for the developmental differences in initial response to alcohol remains speculative. Recent evidence suggests, however, that the relative resistance of adolescents to the sedative effects of alcohol is related in part to both accelerated development of acute tolerance (Silveri and Spear 1998; Swartzwelder et al. 1998) and developmental immaturity of the GABA (Silveri and Spear 2002; Li et al. 2003) and/or NMDA (Silveri and Spear 2004) receptor systems. The available data on the consequences of longer-term adaptations (i.e., rapid and chronic tolerance) to alcohol’s effects in adolescents are inconsistent, with studies indicating more tolerance in adolescents than adults, similar levels of tolerance, or the appearance of sensitization rather than tolerance after repeated adolescent exposures (see Spear and Varlinskaya 2005).

### Stress, Hormones, Adolescence, and Alcohol Abuse

Late childhood and adolescence are periods marked by dramatic sexual and psychosocial development. Between the ages of 5 and 9, adrenarche occurs, resulting in increased secretion of many adrenal steroids (cortisol, androstenedione, dehydroepiandosterone). Adrenal androgens in humans are associated with auxiliary and pubic hair growth and a slight increase in bone and skeletal growth. This is followed by maturation of the reproductive system, also referred to as “gonadarche,” which is characterized by increased activity of gonadotropins and the sex steroids (estradiol in females and testosterone in males). The hypothalamic-pituitary-adrenal axis response to stress also undergoes development during the pubertal period (e.g., Goldman et al. 1973; Sapolsky et al. 1985). Increased life stressors associated with sexual and social maturation, together with hormonally induced mood and behavior changes, could contribute to increased consumption of alcohol during the adolescent period (Tschann et al. 1994).

In adult humans and animals, the relationships among stress, drinking, and underlying neuroendocrine or neurochemical mechanisms are complex. However, basic animal research suggests that stress-induced changes in glucocorticoids (i.e., corticosterone) may interact with neurotransmitters in the mesolimbic reward system to facilitate drinking. In adolescents, the interaction between stress and drinking is even more complicated because the neural systems involved in modulating alcohol reward and/or stress are undergoing development (see Spear 2000 for review).

In a few studies of adolescent non-human primates, it has been shown that under conditions of social separation stress, subjects double their rates of alcohol consumption (Higley and Linnoila 1997*b*; Fahlke et al. 2000). In these studies, individual differences in stress-induced drinking are attributed to anxietylike behaviors mediated by ontogenetic changes in cortisol and corticotropin levels or to poor impulse control and impaired social competence associated with reduced serotonin functioning (a traitlike marker present in infancy).

In rats, findings indicate that adolescent animals exhibit an attenuated corticosterone response to alcohol challenge compared with adults, and that gender differences in this response begin to emerge at adolescence (Ogilvie and Rivier 1996; Silveri and Spear 2004; for review, see Spear 2000). If elevations in corticosterone contribute to the rewarding effects of alcohol, as indicated in adult animals, then adolescents may need to increase their levels of alcohol to attain the reinforcing value reached by more mature animals at lower levels. At this point, however, any hypothesized interactions between stress-induced changes in hormones and reward-related neurotransmitters, and their impact on adolescent drinking, remain tentative.

Gonadal hormones influence many aspects of brain development and behavior outside the realm of reproductive functions via their actions on receptors located throughout the brain (see Wang et al. 2002; McEwen 2002 for reviews). Alcohol’s disruptive effects on pubertal hormone secretion may interfere with hormonally mediated developmental processes and result in negative behavioral outcomes. For example, alcohol-induced increases in testosterone are related to augmented aggression in male hamsters following chronic alcohol exposure during adolescence (Ferris et al. 1998). Evidence from adult female nonhuman primates indicates that sensitivity to the subjective effects of ethanol changes during different phases of the menstrual cycle as a result of alterations in endogenous levels of ovarian-derived hormones (Grant et al. 1997). However, virtually nothing is known about adolescent females’ sensitivity to the subjective or aggressive effects of alcohol or the correlation of these effects with cyclical hormonal changes. Given that adolescence is a time when hormonal and brain systems are still developing in humans and animals, research on the relationships among life stressors, affective states, and hormonal/neurotransmitter interactions may be critical to understanding the onset, maintenance, and consequences of adolescent drinking.
